# Correction

**DOI:** 10.1080/28324765.2025.2457879

**Published:** 2025-01-28

**Article title**: Mental health self-stigma: links with social self-worth contingencies and ally support

**Authors**: Rebecca A. Burwell and Sierra Bias

**Journal**: Cogent Mental Health

**DOI**: 10.1080/28324765.2024.2310039

This article was originally published with an error in the text. To rectify this, the following additional content has now been included at the end of the *Data analytic approach*.

This study was approved by the Westfield State University Institutional Review Board (IRB Number 17/18-032).

